# The mitochondrial genome of *Desmomyia sinensis* (Diptera: Rhagionidae)

**DOI:** 10.1080/23802359.2021.1934166

**Published:** 2021-06-03

**Authors:** Yue Liu, Jingyu Wang, Ding Yang

**Affiliations:** Department of Entomology, College of Plant Protection, China Agricultural University, Beijing, China

**Keywords:** Mitochondrial genome, Tabanomorpha, Rhagionidae, phylogenetics

## Abstract

The mitochondrial genome of *Desmomyia sinensis* Yang et Yang, 1997 was sequenced, the new representative of the family Rhagionidae. The complete mitochondrial genome is 16,430 bp totally, which consists of 13 protein-coding genes, two ribosomal RNAs, 22 transfer RNAs, and one non-coding control region. The nucleotide composition is biased toward A and T, accounting for 77.9% of the total. All genes have the conservational arrangement like other published species of Rhagionidae. Bayesian inference analysis strongly supported the monophyly of Rhagionidae and sister relationship between Coenomyiidae and Xylophagidae. The phylogenetic relationship among families of Tabanomorpha is very clear: (Athericidae + Tabanidae) + (Rhagionidae + (Coenomyiinae + Xylophagidae)).

The family Rhagionidae belongs to Tabanomorpha of lower Brachycera (Diptera) with 28 genera and over 700 species known from the world. Members of the family are small to medium-sized with hairs but no obvious bristles. Rhagionids have diverse habitats. Larvae of rhagionids are usually found in moist soil rich in decaying organic matter, liverwort, and moss, as well as on the edges of streams and running water (Yang et al. 2016). Most adults appear on the forest edge and vegetation beside lakes, ponds, reservoirs, and streams (Yang et al. 2016). Some species of Rhagionidae have the habit of sucking blood (Knab and Cooley 1912; Aldrich 1915; Wirth 1954). The genus *Desmomyia* is a rare group in the family Rhagionidae with just two known species, which are only distributed in the Oriental region (Yang et al. 1997).

The specimens of *D. sinensis* (voucher number: CAUYDRHAG-Desm-1) used for this study were collected from Jitai Valley (28°40′ N, 97°31′ E, 3296 m), Chayu, Tibet, on 4 June 2020. The specimens were identified by Ding Yang through the following characters: head, thorax, and abdomen all black with pale gray pollinose; mesonotum with three darker wide longitudinal spots, of which median one is divided by a pale line; wing brown with several paler parts; all femora dark brown to black, but all tibiae brownish yellow to yellow except tips in both sexes (Yang et al. 1997). Specimens are deposited in the Entomological Museum of China Agricultural University (CAU).

The genomic DNA was extracted from adult’s thoracic muscle tissues using the DNeasy DNA Extraction kit (TIANGEN, Beijing, China) and stored at −20 °C. The sequencing followed the procedures of Gillett et al. (2014), the pooled DNA sample was sent to BIONONA Co., Ltd. (Auckland, New Zealand) for library construction using the TruSeq chemistry with an insert size of 480 bp. The library was sequenced on a single Illumina HiSeq 2500 lane with 500 cycles of paired-end sequencing (250 bp reads). After removing adapters, unpaired, short and low quality reads using Trimmomatic v0.30 (Bolger et al. 2014), 4 GB of high-quality, filtered reads were used for *de novo* assembly with Meta-IDBA (Peng et al. 2012). The bait sequence *COI* was amplified by standard PCR reactions, and BLAST search was carried out with BioEdit 7.0.5.3 (Hall 2011). The assembled mitochondrial genome was first uploaded as the original Fasta file to MITOS (Bernt et al. 2013) to identify open reading frames (ORFs), rRNAs, and tRNAs, and each protein-coding gene was identified manually in MEGA 7.0 by aligning with other Rhagionidae species (Kumar et al. 2016).

For Bayesian inference (BI), we first converted reading frames into amino acids and aligned them in MEGA 7.0 (Kumar et al. 2016), then used Phylosuite to concatenate all these individual alignments into a single matrix (Zhang et al. 2020). We used PartitionFinder2 (Lanfear et al. 2016) to search for the best-fitting scheme, and used Bayesian information criterion (BIC) to estimate the optimal nucleotide substitution model for each partition. We used the best-fit partitioning scheme and partition-specific model recommended by PartitionFinder2 (Lanfear et al. 2016), and used MrBayes 3.2.7a (Ronquist et al. 2012) on CIPRES (Miller et al. 2011) for inference.

The complete mitochondrial genome of *D. sinensis* (GenBank accession number: MW394519) is 16,430 bp in length and consists of 13 PCGs, 22 tRNA genes, two rRNA genes, and one non-coding control region, which are similar to *Rhagio* sp. (Wang et al. 2016). The nucleotide composition of the mitochondrial genome is biased toward A and T, with 77.9% of A + T content (A = 40.0%, T = 37.9%, C = 12.9%, G = 9.2%). Among the protein-coding genes, three PCGs (*NAD2*, *NAD3*, and *NAD5*) initiate with ATT codons, six PCGs (*COII*, *COIII*, *ATP6*, *NAD4*, *NAD4L*, and *CYTB*) initiate with ATG codons, two PCGs (*ATP8* and *NAD6*) initiate with ATC codons, *NAD1* initiates with ATA as a start codon, and *COI* initiates with CCG as a start codon, respectively. Eleven PCGs use the typical termination codons TAA, one PCG (*NAD3*) uses TAG, and the remaining one (*NAD4*) uses TA in *D. sinensis*.

Phylogenetic trees (Figure 1) were inferred using BI based on the nucleotide sequences of 13 PCGs from 11 Diptera species. *Tipula abdominalis* and *Chironomus tepperi* were chosen as outgroup. The Bayesian inference analysis strongly supported the monophyly of Rhagionidae and sister relationship between Coenomyiidae and Xylophagidae, which were consistent with the previous study (Wang et al. 2016). The phylogenetic relationship among families of Tabanomorpha is very clear: (Athericidae + Tabanidae)+(Rhagionidae+(Coenomyiidae + Xylophagidae)). The complete mitochondrial genome of *D. sinensis* may provide valuable information for future genetic and evolutionary studies of Rhagionidae and Tabanomorpha.

**Figure 1.**
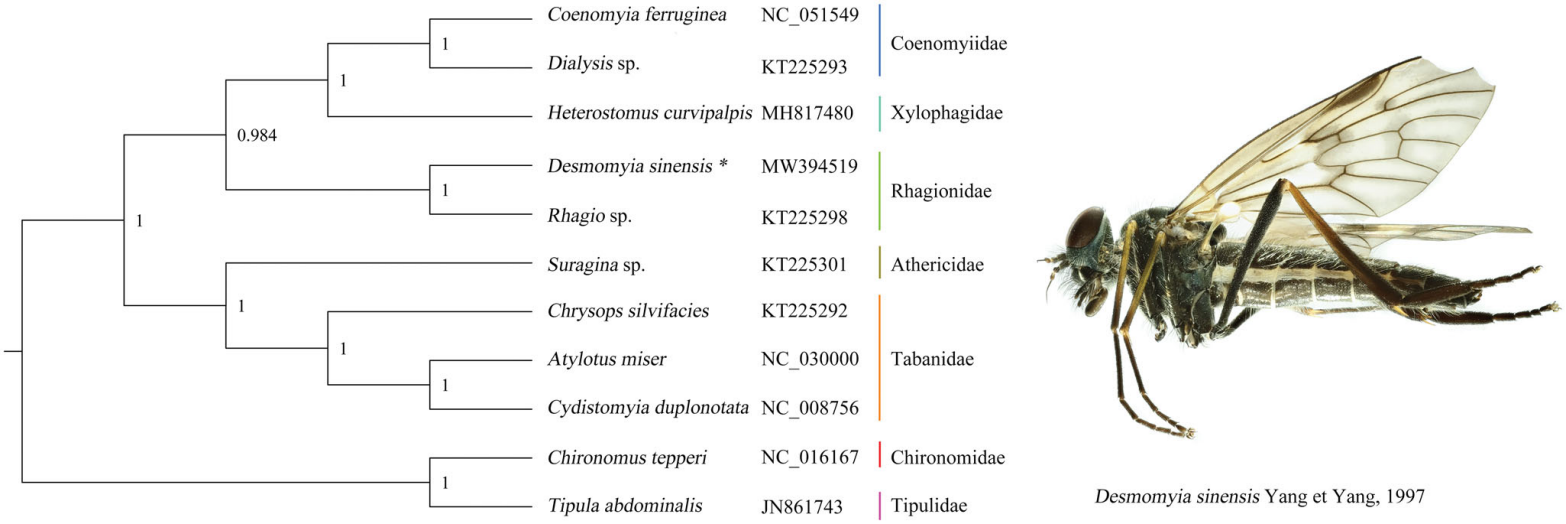
The phylogenetic tree of BI analysis based on 13PCGs, and adult of *Desmomyia sinensis* Yang et Yang, 1997. *New sequenced data in this study. Bayesian posterior probabilities were labeled at each node. The GenBank accession numbers for all species: *Coenomyia ferruginea* (NC_051549); *Dialysis* sp. (KT225293); *Heterostomus curvipalpis* (MH817480); *Desmomyia sinensis* (MW394519); *Rhagio* sp. (KT225298); *Suragina* sp. (KT225301); *Chrysops silvifacies* (KT225292); *Atylotus miser* (NC_030000); *Cydistomyia duplonotata* (NC_008756); *Chironomus tepperi* (NC_016167); *Tipula abdominalis* (JN861743).

## Data Availability

The data that support the findings of this study are openly available in NCBI at https://www.ncbi.nlm.nih.gov/MW394519. Associated BioProject, https://www.ncbi.nlm.nih.gov/bioproject/PRJNA706850, BioSample accession number at https://www.ncbi.nlm.nih.gov/biosample/SAMN18146016, and Sequence Read Archive at https://www.ncbi.nlm.nih.gov/sra/ SRR13908658.
